# Small cell osteosarcoma of a toe phalanx: a case report and review of literature

**DOI:** 10.1186/1749-799X-5-36

**Published:** 2010-06-03

**Authors:** Jantine PosthumaDeBoer, Harm CA Graat, Johannes Bras, Rachid Saouti

**Affiliations:** 1 Department of Orthopaedic Surgery, VU University Medical Centre, Amsterdam., The Netherlands; 2 Department of Pathology, Academic Medical Centre, Amsterdam, The Netherlands

## Abstract

This report describes the radiological and histological findings of a small cell osteosarcoma of a toe phalanx in a 38 year old man. This man presented with pain, swelling and redness of the left third toe. Medical history revealed an osteomyelitis of this toe eight years prior. Based on clinical findings and medical history the lesion was diagnosed as an osteomyelitis. However, peroperatively the lesion had a malignant aspect. Histological examination revealed a small cell osteosarcoma of the proximal phalanx.

Osteosarcoma of the foot and especially of the tubular bones is rare. Moreover small cell osteosarcoma is a rare subtype of osteosarcoma. This case demonstrates that medical history and clinical examination can be misleading. In patients with apparent bone destruction, a malignancy must always be excluded prior to treatment. It emphasises the care that should be taken in the process of formulating a diagnosis.

## Background

Osteosarcoma (OS) is the most common primary malignant bone tumor in children and adolescents with a peak incidence at age 15-19 years old. Typically, it originates from the metaphysis of long bones and has a high tendency to systemic spread [1].

Osteosarcoma of short tubular bones is a rarity [2-5]. An osteosarcoma of a toe phalanx was first ever described by Mirra et al in 1988 [6,7]. Since then only a few cases have been reported.

Osteosarcoma in the foot represents approximately 0,5% of all osteosarcomas [1,3]. As opposed to conventional osteosarcoma it is more often encountered in adult patients [4,8]. A higher percentage of osteosarcoma of tubular bones is found to be low grade thus less aggressive than osteosarcomas in general [2,3,9]. Interestingly, this case represents a small cell osteosarcoma, which is an aggressive subtype.

Small cell osteosarcoma, first described in 1979 by Sim et al [10], classifies as a medullary osteosarcoma according to the WHO's histologic classificiation (World Health Organisation) of bone tumors[11]. It is a rare, though distinct subtype of osteosarcoma with an incidence rate of approximately 1.3% of all osteosarcomas [6,8,12,13]. It has the same distribution concerning age and skeletal location as conventional osteosarcoma. The treatment of small cell osteosarcoma is also similar to that of conventional osteosarcoma, consisting of a multi-agent chemotherapy regimen and surgery [6].

To the best of our knowledge, this is the first case of small cell osteosarcoma originating from a phalanx presented in English-language literature.

## Case Report

**Figure 1:**
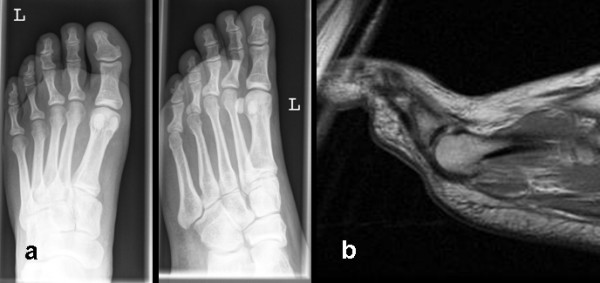
**This figure shows anterioposterior and 3/4 (A) conventional radiographs and a T1 weighed MRI section in the sagittal plane (B) of the third toe of the left foot**. **A**. There is a small fracture at the base of the proximal phalanx of the third toe. **B**. This MRI section shows an intra-osseous lesion, without expansion beyond the cortical border of the phalanx.

In the year 2000, a 30 year old, otherwise healthy male presented with pain, swelling and redness in his left third toe. Radiological findings showed a pathological fracture of the proximal phalanx of the third toe on the basis of an intra-osseous lesion with a spotted and sclerotic aspect. An MRI was made showing a clear abnormality of the proximal phalanx of the third toe with oedema of the bone marrow and minimal reaction in the adjacent soft tissue. There was no extra-cortical expansion. Osteomyelitis or Ewing's sarcoma could not be excluded. (Fig. 1)

In January 2001 a biopsy was performed. Histopathology showed cortical and cavernous bone tissue with reactive changes and damaged cells of which identification was not possible. The bone abnormalities were considered to be reactive in nature and the diagnosis was set as osteomyelitis.

The patient's complaints diminished spontaneously. No antibiotic treatment was prescribed. In April 2001, a control radiograph showed a normal structure of the third proximal phalanx of the left foot. The patient was discharged from follow-up in the outpatient clinic.

At the time of the second presentation, in 2008 with the patient being 38 y, the same symptoms occurred. They were present since six weeks. Physical examination showed clear swelling and tenderness of the left third toe. There was no ulceration.

**Figure 2:**
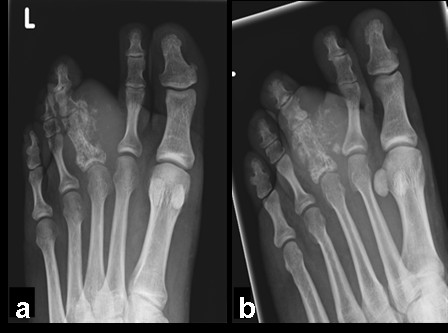
**This figure shows anterioposterior (A) and 3/4 (B) conventional radiographs of the left foot**. There is a diffuse lesion of the proximal phalanx of the third toe with a moth eaten aspect, cortical destruction and calcifications in the soft tissue.

The conventional anteroposterior radiograph (Fig. 2) of the left foot showed a moth-eaten aspect of the proximal phalanx of the third toe with cortical destruction. There is an infiltration in, and swelling of the soft tissue surrounding the lesion. The head of the third metatarsal bone is intact.

The total-body bone scintigraphy showed high uptake of TC-99M in the left third toe and was suggestive of osteomyelitis. There were no other hotspots.

The clinical diagnosis was set at osteomyelitis and the patient was surgically treated as such. In august 2008 the proximal phalanx of the left third toe was surgically removed. Macroscopically there was a large mass in the surrounding soft tissue with calcifications. The findings were highly suggestive for a malignancy. Due do the initial diagnosis of osteomyelitis the surgical margins were intralesional.

### Histological examination

**Figure 3:**
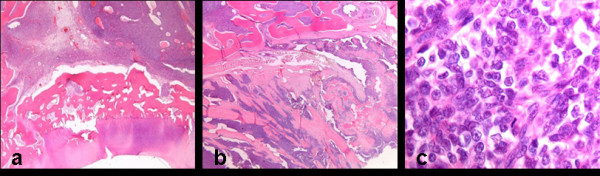
**The histological specimens show typical characteristics of a small cell osteosarcoma**. **A**. The cartilaginous surface of the proximal phalanx of the third toe at the side the metatarso-phalangeal joint. A vast field of purple tumor cells underneath the cartilage, without infiltration of the cartilage. There are atypical small cells which lie in a compact fashion. (HE staining, 1,25× objective) **B**. A cross section of the outer cortex of the proximal phalanx. The tumor comprises fields of many small purple cells. The cortex is disrupted totally by the atypical cells. The tumor is situated in and around the cortex of the phalanx. The cells infiltrate the surrounding soft tissue, where there is osteoid formation by the tumor cells. This feature defines this tumor as being an osteosarcoma. The osteoid formation was in a lamellar fasion. Within the formed osteoid the same atypical cells are present. (HE staining, 1,25× objective) **C**. A close up of the tumor cells. It shows typical small and anaplastic, poorly differentiated cells with dark, hyperchromatic nuclei and very little cytoplasm. (HE staining, 63× objective)

Histological examination followed.  Microscopically there are large fields of atypical small blue round cells. The cells are highly undifferentiated and are very uniform in appearance. The nuclei are dark and round to oval, there is little cytoplasm. There is osteoid formation by the tumor cells. (Fig. 3) The pathological diagnosis is small cell osteosarcoma of the phalanx.

### MRI

**Figure 4:**
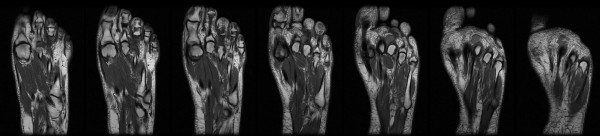
**This figure shows a series of T1 weighed sections in the axial plane of the left foot**. There is an increase in signal in the soft tissue at the former site of the third proximal phalanx which was reasoned to be partially due to the surgery and wound healing. A small oval lesion was detected between the heads of the second and third metatarsal bone. This was interpreted as a residual malignant lesion.

Metastatic disease was not found on a CT-scan of the thorax and abdomen.  An additional MRI of the left foot was made in September 2008. This showed the initial post-operative situation. (Fig 4)

From November 2008 the patient was treated according to the EURAMOS-1 trial [11]. In February 2009 additional surgery was performed. The third and second rays of the left foot were amputated. The histological specimen showed one small residual lesion in the soft tissue of the second web space. There was a good response to induction chemotherapy as shown histologically by a high rate of necrosis within the tumor. The patient was randomised in the EURAMOS-1 trial and treated with 4 cycles of Adriamycin and Cisplatinum. Methotrexate was abandoned after 2 cycles due to severe mucositis. At time of submission of this paper, the patient is living and well. At 6 months follow-up there are no signs of recurrent of systemic disease.

In retrospect, the initial abnormalities in 2001 are verified as infectious by the pathologist.

## Discussion

Small cell osteosarcoma is rare and relatively little English-language literature is available on this subject. Most publications comprise case reports.

The clinical and radiological features resemble those of the conventional osteosarcoma. The most common clinical features of small cell osteosarcoma are pain and swelling [10]. Often, symptoms are mild so that there is a delay in the first visit to a physician. Misdiagnosis is common because the clinical findings are highly non-specific.

On radiographs small cell OS shows lesions with either lytic or sclerotic features, or a combination of the two [3,6,10,14,15]. Edeiken et al. [13] suggest that the mixed pattern of lytic and sclerotic areas is suspect for small cell rather than conventional OS. Often cortical destruction and calcifications in surrounding soft tissue are present. New bone formation by the tumor excludes the diagnosis of Ewing's sarcoma [13]. On conventional radiographs small cell OS, as well as conventional OS, can mimic other lesions of the bones such as an aneurismal bone cyst, osteomyelitis, osteoblastoma, chondroblastoma or osteoid osteoma. Reactive bone formation due to chronic inflammation can be mistaken with tumorous bone formation and vice versa on radiography [16]. We advocate the use of a biopsy to assure the diagnosis.

Small cell OS is a distinct subtype of conventional osteosarcoma with its own histological entity. Typically small cell OS shows a uniform population of small, round cells with little cytoplasm and indistinct cell borders. The nuclei are round, occasionally oval shaped. Osteoid production is always present [6,8,10,12,13,15]. This feature is pathognomonic for OS in general [13]. In the diagnosis of small cell OS osteoid formation is a very important feature since it is not seen in Ewing's sarcoma [8,12,13,15]. It is important to distinguish between small cell OS and Ewing's sarcoma because there are different treatment modalities for these two types of tumor [10].

Small cell osteosarcoma is considered an aggressive subtype of OS with a poor prognosis [8,10,12,17].

The Rizolli institute has described clinical, radiological and histological characteristics of small cell OS in a group of patients [12]. Radiological findings include involvement of both medulla and cortex, with disruption of the latter and the presence of a mass in the adjacent soft tissue. Histologically the production of osteoid is present in all tumors. The prognosis of the Rizolli group of patients was poor with only 1 out of 9 patients alive 24 months after diagnosis, regardless of location, surgical and adjuvant therapy. Nakajima et al. [8] states the cumulative 5 y survival is 28,5%, whereas the 5 y survival for conventional osteosarcoma is 65%. These figures illustrate the poor prognosis for this subtype of OS. Mortality is mostly encountered as a result of metastatic disease.

The pattern of metastasis is concordant to that of conventional OS. Metastases are most commonly found in the lungs [15], although it has the potential to metastasize to other skeletal locations also [17].

The treatment of small cell OS is similar to the conventional OS treatment [6,10]. It comprises neo-adjuvant chemotherapy, ablative surgery (if feasible) and adjuvant chemotherapy. The prognosis partly relies on the tumor response to induction chemotherapy.

Apart from the rarity of small cell OS, the patient in this case suffered from an OS on a rare skeletal location. In the literature available on osteosarcoma of the tubular bones (mostly of the hand), it is noted that osteosarcomas of the digits more commonly low-grade [9]. Most osteogenic lesions in the small bones of hands and feet are benign, usually reactive in nature [4].

Since OS of the phanlanges is such a rarity, late or misdiagnosis is common, as was the case in this patient.

It is extraordinary that this patient developed an osteosarcoma at the exact same location where he previously suffered an osteomyelitis. This poses the question whether there is a connection between the two ailments.

According to literature, it is unclear whether osteomyelitis is an etiological factor in the development of osteosarcoma. Although malignancies arising from a draining infection are relatively common, malignant degeneration of chronic, draining osteomyelitis into a sarcoma is seldom [18]. Most malignancies arising in a sinus tract are squamous cell carcinomas [19]. In our case the situation is different as there was no draining osteomyelitis present. There are groups that do describe chronic osteomyelitis as an etiological factor in osteosarcoma [3,20]. Too many uncertainties are present in our case to state that there was a chronic osteomyelitis that degraded into osteosarcoma. The course of the osteomyelitis was somewhat peculiar as the complaints diminished spontaneously. In the interval between the two conditions the patient suffered no complaints whatsoever. To our believe, both ailments have no causal relationship. The previous episode of osteomyelitis did however steer us in a wrong direction setting the diagnosis.

## Conclusion

Osteosarcoma of the phalanges of the foot is extremely rare. Often there is a delay in diagnosis because of patient delay and mildness of symptoms, or because of misdiagnosis at presentation. It affects adults rather than adolescents. In adults with a mixed lytic and sclerotic lesion on the X-ray, an osteosarcoma in the foot should be considered in the differential diagnosis.

This report was written to accentuate the risk of misdiagnosis in the case of a rare disease in a patient with only mild symptoms. A thorough pre-operative work up is always necessary. In the case of a destructive bone lesion, always rule out a malignancy prior to surgical treatment.

## Conflict of interests statement

The authors declare that they have no competing interests.

## Authors' contributions

JPDB: designed manuscript, collected patient information and performed literature search. HCAG: advised on design and corrected manuscript. JB: analysed histological samples, approved manuscript. RSA: supervised project, provided patient information and corrected manuscript. All authors read and approved the final manuscript.

## Consent

Written informed consent was obtained from the patient for publication of this case report and the accompanying images and coupes. A copy of the written consent is available for review by the Editor-in-Chief of this journal.
